# Supplementary material for: Geometric transformation and three-dimensional hopping of Hopf solitons
Source: Nat Commun. 2022 May 27;13:2986. doi: 10.1038/s41467-022-30494-2 (PMC9142506; doi:10.1038/s41467-022-30494-2)
Supplement: Supplementary file 1 — Supplementary Information [file 41467_2022_30494_MOESM1_ESM.pdf]

Supplementary Information for

Geometric transformation and three-dimensional hopping of Hopf solitons

Jung-Shen B. Tai[1], Jin-Sheng Wu[1] and Ivan I. Smalyukh[1,2,3*]

[1]*Department of Physics and Chemical Physics Program, University of Colorado, Boulder, CO 80309, USA*

[2]*Department of Electrical, Computer, and Energy Engineering, Materials Science and Engineering Program and Soft Materials Research Center, University of Colorado, Boulder, CO 80309, USA*

[3]*Renewable and Sustainable Energy Institute, National Renewable Energy Laboratory and University of Colorado, Boulder, CO 80309, USA*

* *Correspondence to: ivan.smalyukh@colorado.edu*

Supplementary Figures

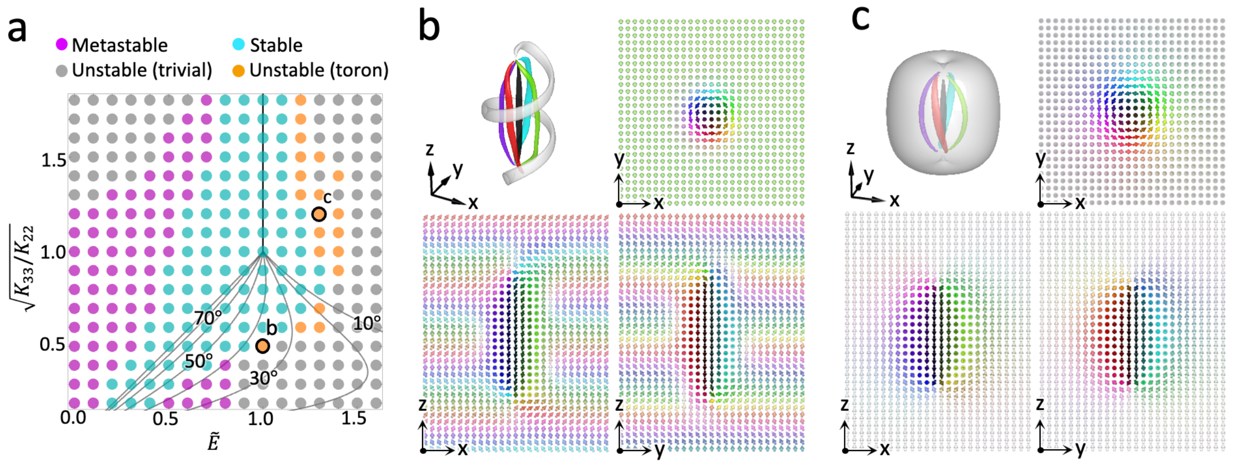

Supplementary Fig. 1 | Numerical simulations of Hopf solitons in the chiral liquid crystal (LC) bulk. a, Structural stability diagram of Hopf solitons in the chiral LC bulk as shown in Fig. 2a but here additionally distinguishing torons and states with trivial topology in the unstable regions. The θ_c contour lines of the embedding background are shown on the diagram. $\boldsymbol{n}(\boldsymbol{r})$ of labeled data points in (a) are shown in (b-c). **b-c**, Preimages and $\boldsymbol{n}(\boldsymbol{r})$ in different cross-sections of torons in a conical background (b) and a uniform background (c) with corresponding parameters labeled in (a).

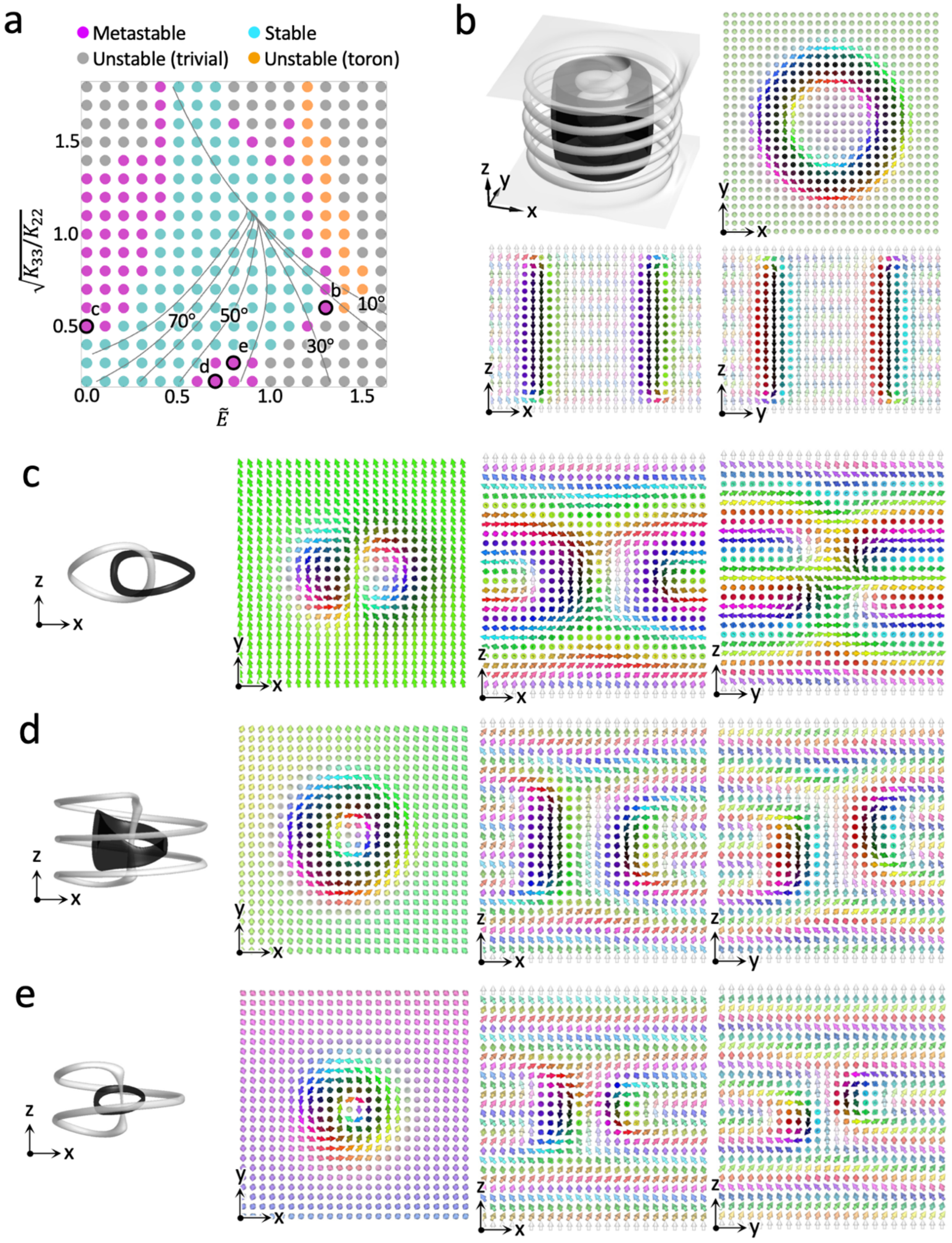

Supplementary Fig. 2 | Numerical simulations of Hopf solitons in chiral LCs with confinement and perpendicular boundary condition (BC). a, Structural stability diagram of Hopf solitons in a confined chiral LC with perpendicular BC as shown in Fig. 2b but here additionally distinguishing the stability of torons and states with trivial topology in unstable regions. $\boldsymbol{n}(\boldsymbol{r})$ of labeled data points in (a) are shown in (b-e). **b-e**, Preimages and $\boldsymbol{n}(\boldsymbol{r})$ in different cross-sections of Hopf solitons with corresponding parameters labeled in (a).

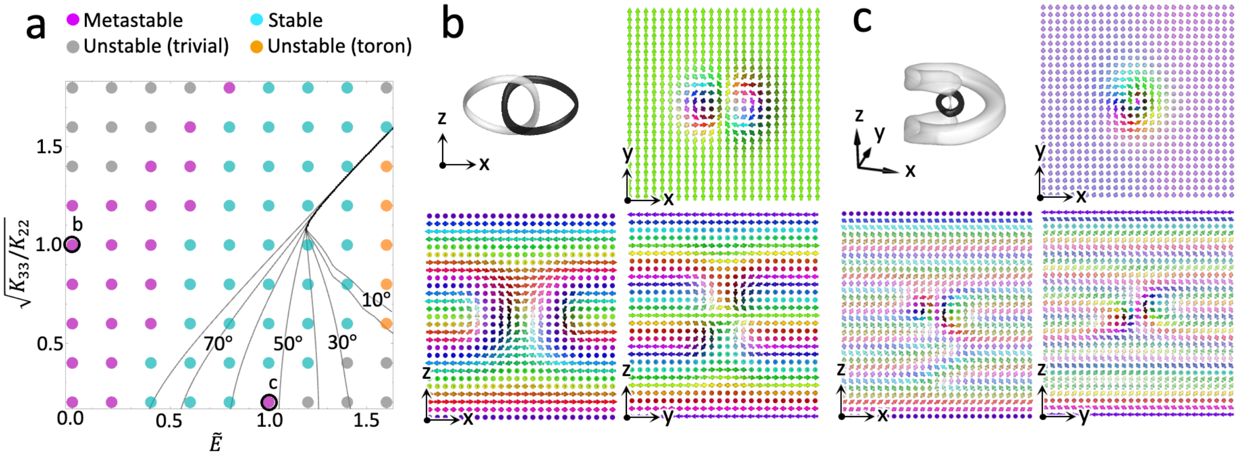

Supplementary Fig. 3 | Numerical simulations of Hopf solitons in chiral LCs with confinement and parallel BC. a, Structural stability diagrams of Hopf solitons in a chiral LC with confinement and parallel BC. Colors represent the stability of Hopf solitons, with torons distinguished from smooth topologically trivial backgrounds. $\boldsymbol{n}(\boldsymbol{r})$ of labeled data points in (a) are shown in (b-c). **b-c**, Preimages and $\boldsymbol{n}(\boldsymbol{r})$ in different cross-sections of Hopf solitons with corresponding parameters labeled in the stability diagram in (a).

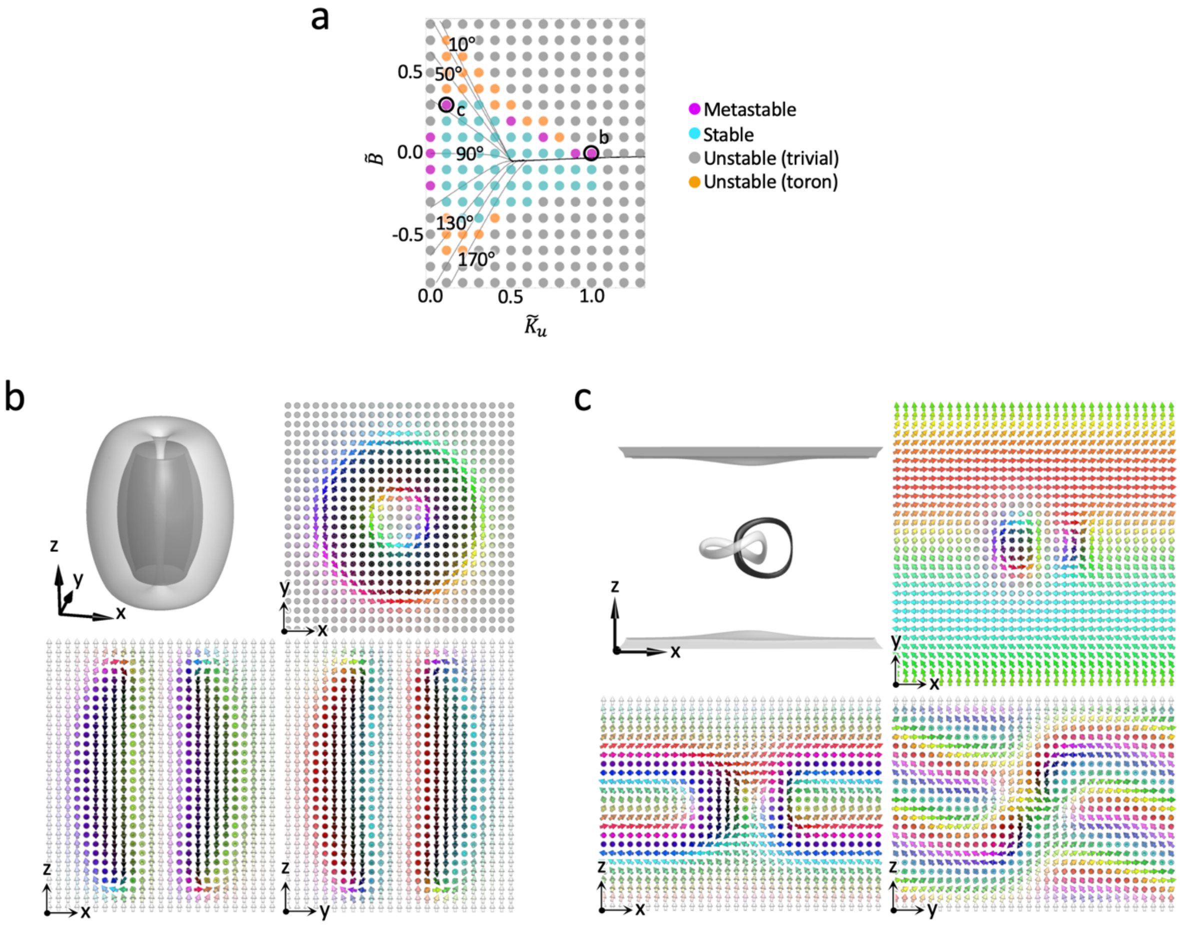

Supplementary Fig. 4 | Numerical simulations of Hopf solitons in confined chiral magnets. a, Structural stability diagram of Hopf solitons in a confined chiral magnet with perpendicular BC achieved by interfacial perpendicular magnetic anisotropy as shown in Fig. 2c but additionally distinguishing torons and states with trivial topology in unstable regions. Colors represent the stability of Hopf solitons, with torons distinguished from smooth topologically trivial backgrounds. $\boldsymbol{m}(\boldsymbol{r})$ of labeled data points in (a) are shown in (b-c). **b**, Preimages and $\boldsymbol{m}(\boldsymbol{r})$ in different cross-sections of a hopfion with parameters corresponding to the labelled data point in (a). **c**, Similar visualizations of a heliknoton with parameters corresponding to the labeled data point in (a).

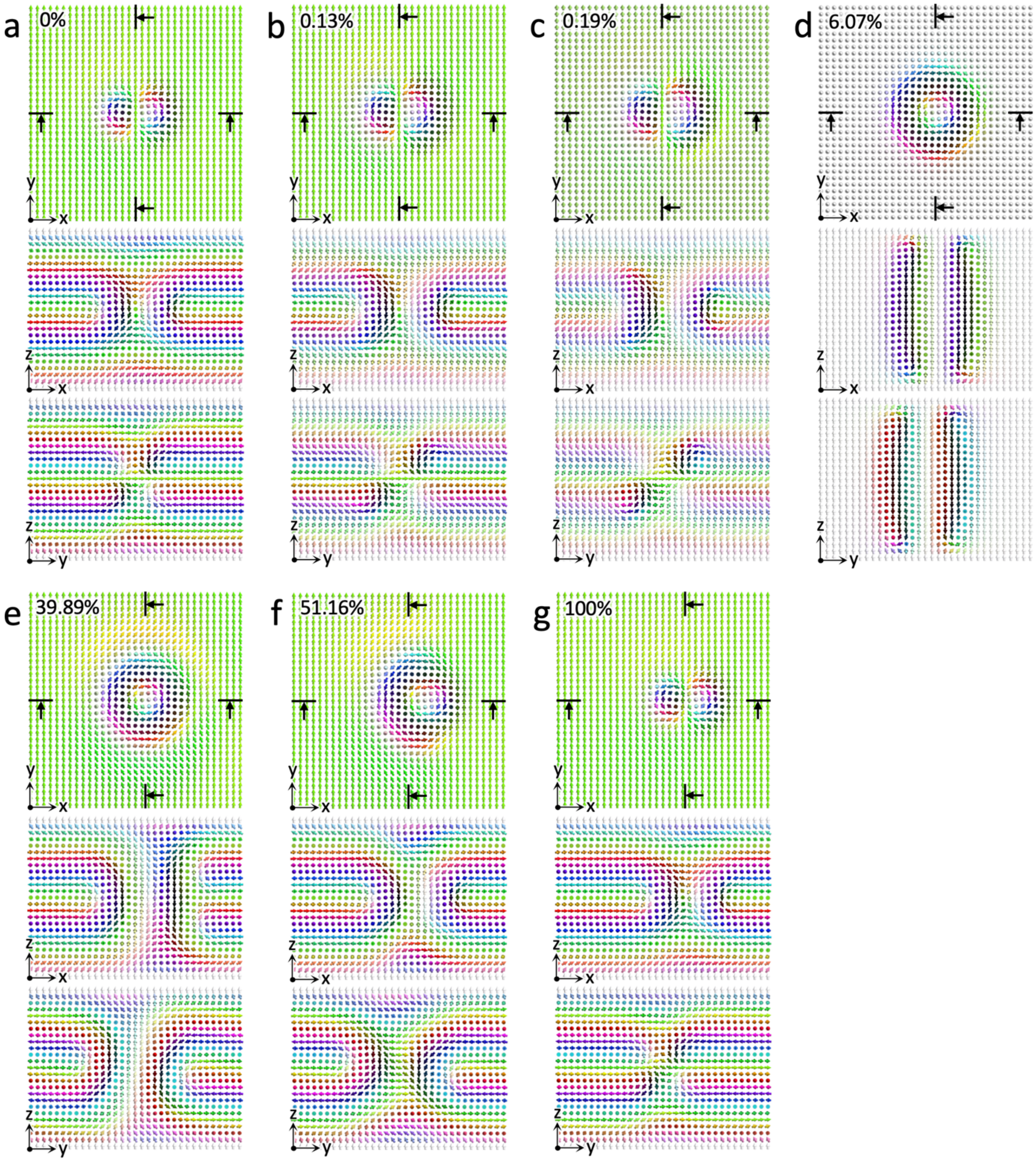

Supplementary Fig. 5 | Simulated cross-sections of the geometric inter-transformation of a Hopf soliton. a-g, $\boldsymbol{n}(\boldsymbol{r})$ of a transforming Hopf soliton corresponding to Fig. 4f-g, shown in orthogonal cross-sections. The xy-cross-sections go through the midplane of the computational volume and the positions of vertical cross-sections (xz and yz) are labeled.

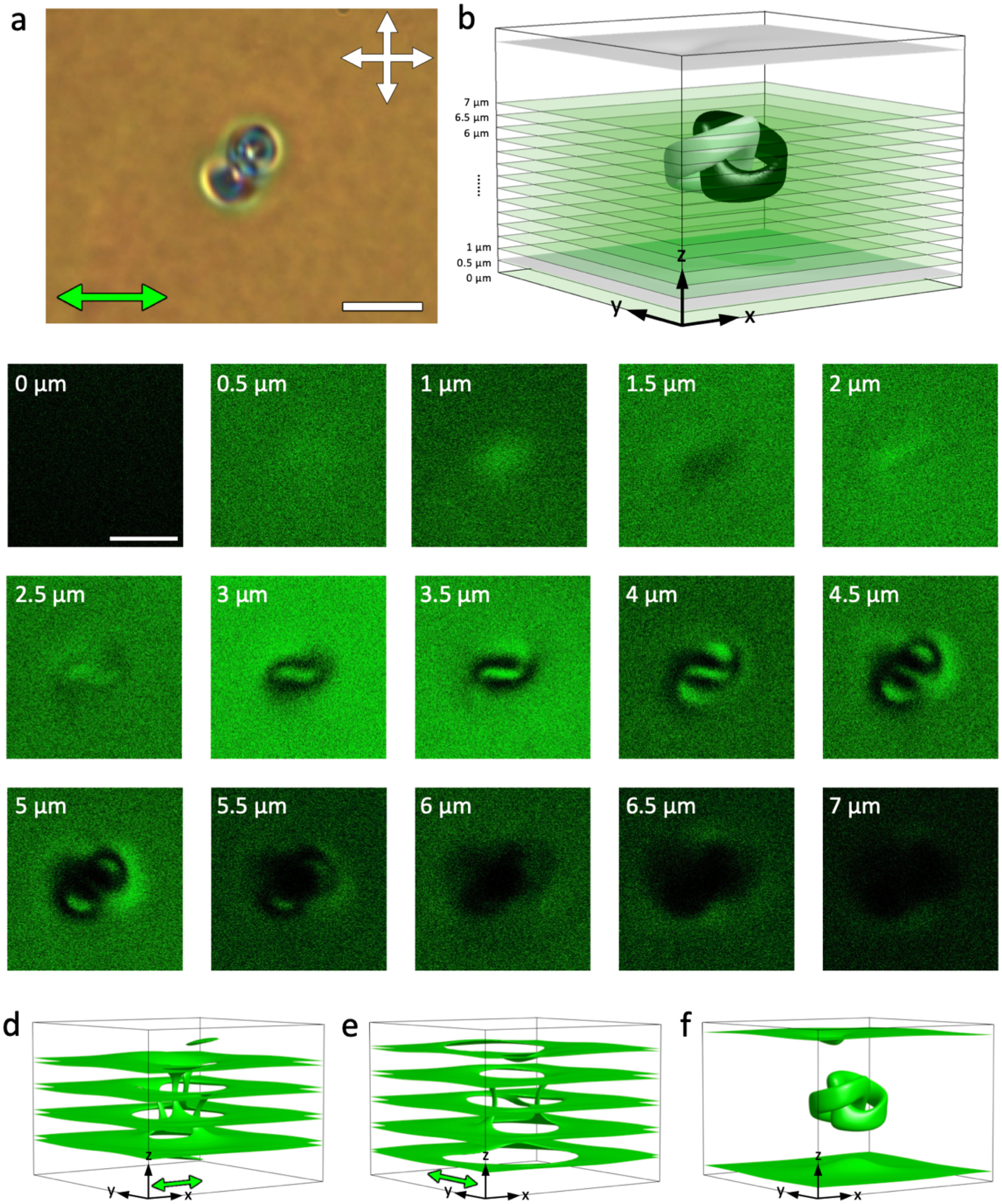

Supplementary Fig. 6 | Three-photon excitation fluorescence polarizing microscopy (3PEF-PM) images of a Hopf soliton. a, Polarizing micrograph of a Hopf soliton in a helical background with confinement and perpendicular BC. **b**. Schematic illustration of the vertical sectioning of 3PEF-PM images through a Hopf soliton shown by preimages of $\pm\hat{z}$ vector orientations. **c**, 3PEF-PM images of a Hopf soliton in horizontal cross-sections shown in (b) with the polarization direction of the excitation light shown in the lower-left of (a). **d-f**, Simulated 3PEF-PM images of a Hopf soliton in a helical background with confinement and perpendicular BC excited by linearly polarized (d & e, polarizations labeled) and circularly polarized (f) femtosecond laser illumination. $d = 3p_0$, $d = 7$ μm and scale bars are 5 μm.

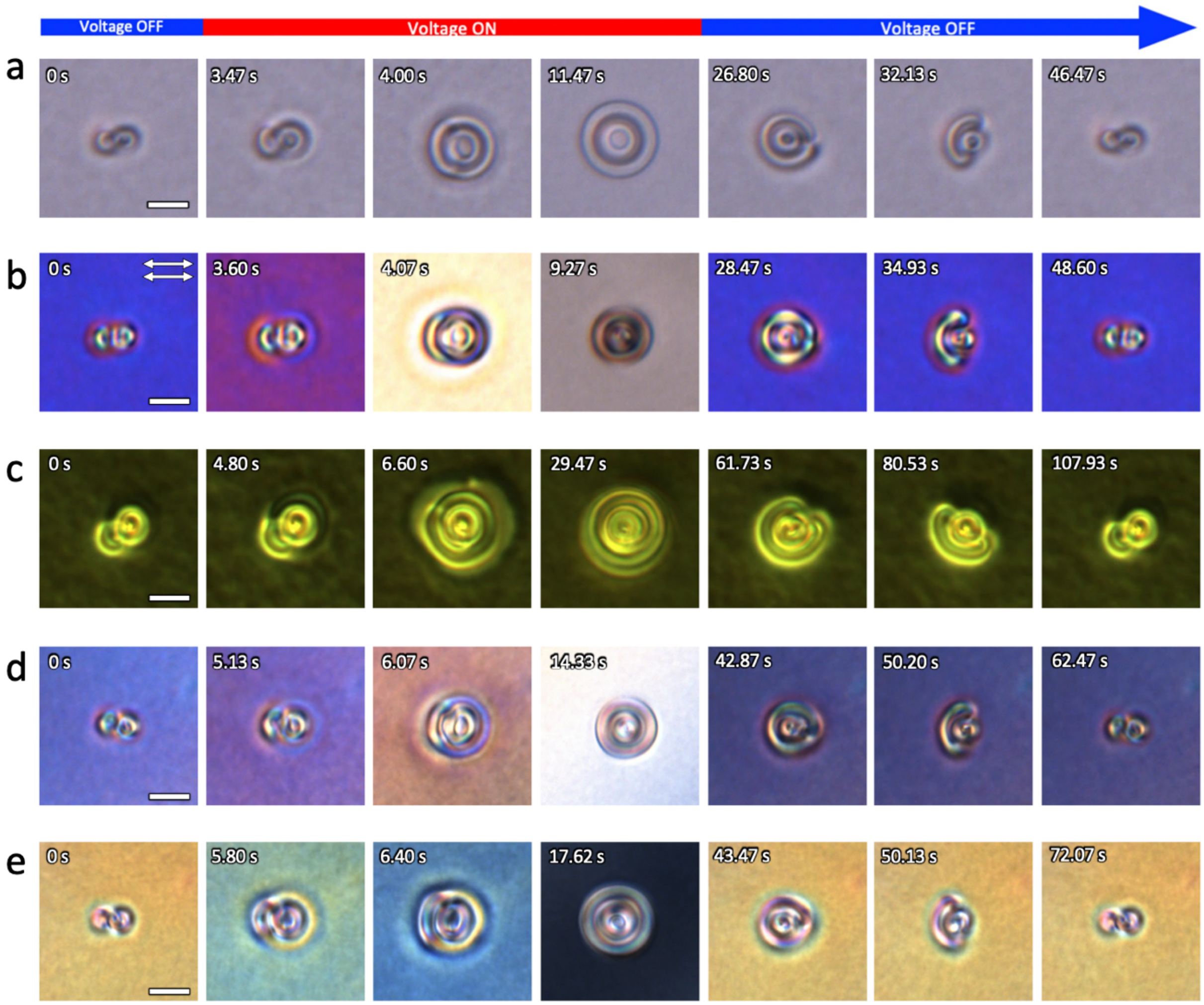

Supplementary Fig. 7 | Geometrical inter-transformation of Hopf solitons observed by different optical imaging modalities. The structures correspond to those shown in Fig. 4. **a**, Bright-field imaging. **b**, Polarizing optical microscopy (parallel polarizers). **c**, Phase contrast microscopy. **d-e**, Differential interference contrast microscopy images obtained for two different Nomarski prism positions. Scale bars are 5 μm.

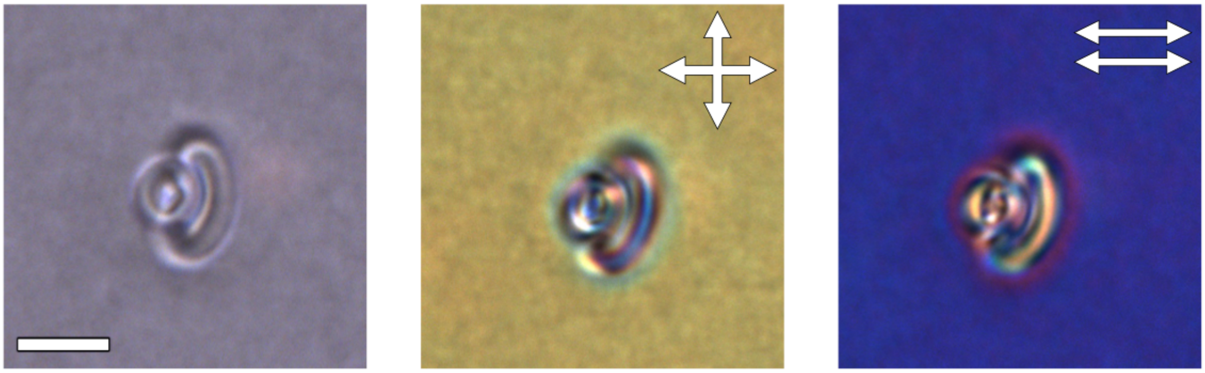

Supplementary Fig. 8 | Hopf soliton stabilized in the conical background. A Hopf soliton in the conical background stabilized at U = 1.76 V is shown in bright-field and polarizing optical micrographs (crossed and parallel polarizers; labeled top right). $d = 3p_0$, $d = 7$ μm and scale bar is 5 μm.

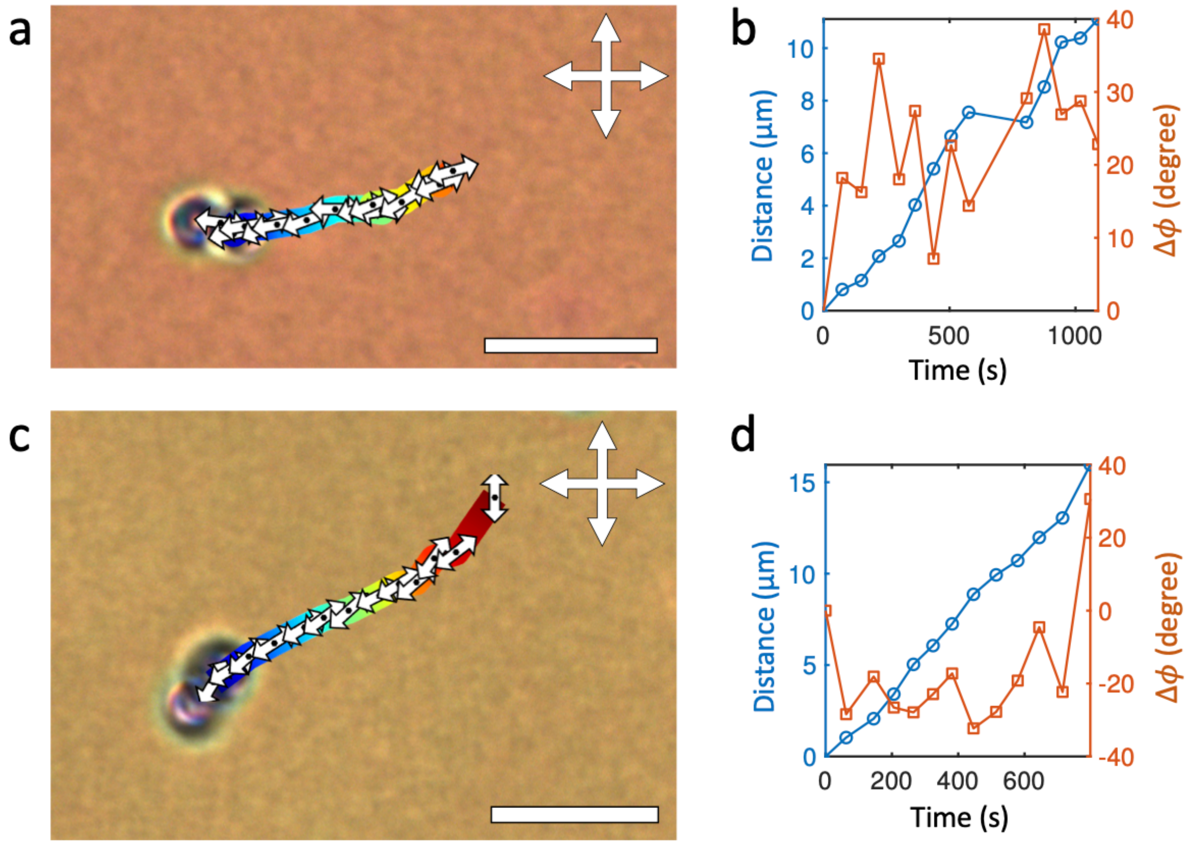

Supplementary Fig. 9 | Additional details of hopping and propelling of Hopf solitons. a-d, Extra data of hopping of Hopf solitons by electric switching shown by the polarizing optical micrographs of solitons at its initial position with 2D color-coded trajectories and orientations shown by double arrows (a,c) and the corresponding distance and accumulated change in orientation in each transformation cycle ($\Delta\phi$) (b,d). Scale bars are 10 μm.
