# Supplementary material for: Geometric transformation and three-dimensional hopping of Hopf solitons
Source: Nat Commun. 2022 May 27;13:2986. doi: 10.1038/s41467-022-30494-2 (PMC9142506; doi:10.1038/s41467-022-30494-2)
Supplement: Supplementary file 3 — Description of Additional Supplementary Files [file 41467_2022_30494_MOESM3_ESM.pdf]

Description of Additional Supplementary Files

Supplementary Movie 1 | Visualizations of the skyrmion number density (blue isosurfaces and green arrows) and vortex lines in the chirality axis field (red) of numerically simulated Hopf solitons in different backgrounds, using the same structures as shown in Fig. 1h-j.

Supplementary Movie 2 | Geometric inter-transformation of a Hopf soliton observed by polarizing optical microscopy videos with crossed polarizers and parallel polarizers (left and middle) and bright-field microscopy video (right).

Supplementary Movie 3 | Geometric inter-transformation of a Hopf soliton observed by phase contrast microscopy.

Supplementary Movie 4 | Geometric inter-transformation of a Hopf soliton observed by differential interference contrast microscopy with two different Nomarski prism positions.

Supplementary Movie 5 | Simulated geometric inter-transformation of a Hopf soliton. The Hopf soliton is visualized by preimages of two antiparallel vector orientations (+z in white and -z in black), and the vertical redline passing through the center of the initial position of the soliton serves as a guide to the eye.

Supplementary Movie 6 | Hopping of a Hopf soliton through repeated inter-transformation between a heliknoton and a hopfion. The voltage was switched between 0 and 3.85 V.

Supplementary Movie 7 | Hopf solitons with +1 and -1 Hopf indices evolving under one full period of voltage modulation. The voltage amplitude varies between 2.27 and 0.07 V with a period of 2 s. Here $d = 1.7p_0$ and $d = 10$ µm.

Supplementary Movie 8 | Squirming motion of Hopf solitons with +1 and -1 Hopf indices under repeated voltage modulations. The voltage amplitude varies between 2.27 and 0.07 V with a period of 2 s. Here $d = 1.7p_0$ and $d = 10$ µm.
